# Report of Discussions before the Ohio State Dental Society, Columbus, December 2d., 1874

**Published:** 1875-02

**Authors:** 


### REPORT OF DISCUSSIONS BEFORE THE OHIO STATE DENTAL SOCIETY, COLUMBUS, DECEMBER 2d., 1874.

Continued from page 52.

---

Dr. Butler: What are the best agents to be employed in covering the pulp?

Dr. Taft: It is impossible, at present, to name the agent which best answers the purpose in all cases. The aim in attempting to preserve the pulp should be to place it as nearly as possible in its original condition. Cover it with some material which will not be decomposed, anything that may be brought in contact with it. It must of course be a non-conductor and a non-irritant. I do not know anything better than the materials which have been suggested. It is important that we have faith to inspire us and a desire and determination to succeed—and a love for the work. The man who does not love his profession can not do justice to those who apply to him for his services. I sometimes hear dentists say "I don't like the practice of dentistry." The sooner such men abandon the profession the better for themselves, the profession, and the public. There is another difficulty which we meet, viz.: the lack of appreciation on the part of the patient. While we are stimulated to put forth our best efforts for those who manifest an interest in our efforts, and who highly appreciate their teeth, who of us has the heart to work for one who cares little whether we succeed or not, who will not consent to suffer inconvenience, or pain if needs be, that the best results may be obtained? Necessarily in such cases there will be failure. The matter of fee or pecuniary reward should not be the controling thought of the dentist in his work. This should be put as far in the background as possible. Let the controling thought be, how can I do the greatest good; success usually brings its reward. There are a few who justly appreciate their teeth. There are a great many who half way appreciate them. There is an innumerable host who care nothing at all about them. They would rather sacrifice their teeth than to suffer pain or even inconvenience for their preservation. In treating the exposed pulp, the manipulations should be very delicately and carefully performed. I think there is fault many times in the manner of manipulation. If the pulp is in condition of health its demand is to be let alone. Prevent the access of irritants to it. I have often filled up the cavity with very finely pulverized charcoal, to neutralize and absorb any noxious gasses. The charcoal will not irritate the pulp if it is very finely pulverized. In regard to the lacto-phosphate of lime treatment I like it very much. I doubt whether it is entitled to the credit which has been claimed for it as an agent in promoting the formation of secondary dentine.

Dr. Watt: What is its modus operandi?

Dr. Taft: I do not know certainly.

Dr. Wells, of Indianapolis: Mr. President, I am not much of a theorist. Am inclined to hold to the old mode of practice—using gutta-percha for capping after securing favorable conditions for the operation. I suppose one reason why I prefer that agent is that I have a tooth filled in that way. I always think of it when I fill over an exposed nerve. One objection which I have to the os-artificiel is the pain which it causes. Have not tried the lacto-phosphate of lime. I admit the importance of saving the pulp. It has been observed by every dentist that the tooth is liable to break down after the pulp is dead; I will agree to all that has been said as to the importance of preserving it. But as to the theory of bringing the pulp into proper condition, that has very little to do with the subject.

Dr. Keely: Perhaps the gentleman will give us his method of using gutta-percha.

Dr. Wells: I take the gutta-peacha in pieces large enough to fill the orifice of exposure entirely. Sometimes I introduce two or three pieces. I warm it so as to make it plastic, and then press it down gently to the nerve. I do not usually hurry the operation. I think it is well, when it is first pressed down to let it cool a little. After it has become somewhat hard I press it against the walls of the cavity with a warm instrument. I am careful not to allow the heat to penetrate to the nerve.

Dr. Jennings: Do you ever experience trouble after this operation?

Dr. Wells: Yes, I take the filling out if there is any pain. I tell the patient to come back the next day. Of course if you should press too hard upon the pulp in introducing the filling you would be likely to have trouble.

Dr. Taft: I must protest against what we are naturally led to infer from the first statement of Dr. Wells. He seems to think that whatever operation can be performed in his own case could be performed in any other. That is a great mistake. He is a man of great vigor and physical strength.

The same operation performed for the majority of persons would probably result in failure. The method is objectionable in two or three respects.

Dr. Watt: I would like to know how lacto-phosphate of lime acts. I want some one to tell us just how it operates. You all know how nitric acid, nitrate of silver, etc., act. I do not like to work in the dark. I have a theory. Correct it if it is wrong. The lactic acid is a solvent of almost all animal tissue. Dead tissue is much more easily dissolved than living tissue. The morbid tissue has less vitality than the normal, and consequently it is much more easily dissolved. In inflammation of an exposed pulp there is plastic matter thrown out, this becomes an irritant to the pulp. I think this the lactic acid dissolves out that vitiated matter. Then if there is putrefaction or disintegration going on, ammonia is one of the results. The lactic acid may neutralize that. All that is merely guess-work with me.

If it is in order, I will tell those who prefer a gutta-percha cap, a mode of preparing it. Take a long phial, fill with chloroform, and drop in shavings of gutta-percha as long as it will dissolve. Let it stand, and there will rise a porous looking matter which contains all the foreign substance. At the bottom you have a whitish looking clear liquid. Save that for use. Keep the phial containing it well corked. In capping the pulp drop a little of this preparation from a blunt instrument, and when the chloroform evaporates you have a cap which will not shrink.

Dr. Horton: Mr. President, I think that Dr. Cravens, who has brought to notice the use of lacto-phosphate of lime, was formerly a pupil of Dr. Watt. In the paper which he read here last year, he told us how he was led step by step to the conclusions at which he arrived finally, in regard to the action and effects of the agent mentioned. I can not now recall the substance of the paper. I do not know whether the gentleman himself would be able to explain its modus operandi. The theory as proposed by Dr. Watt is so beautiful and so captivating, that unto this present moment it is my hope that it may prove correct. I do not think that the practice has advanced far enough yet to enable us to come to any definite conclusion as to its mode of operating. Do not know of any one who is better qualified to correspond with Dr. Cravens on the subject than Dr. Watt.

As to the idea of inducing a deposition of secondary dentine, it is my conviction that if conditions are favorable, and there is nothing in the system—in the blood—to interfere; that the pulp has in its own organization the means of throwing out new dentine for its protection. We find this throughout the whole organization. We find a successful effort made by nature in the case of a wound made in the tusk of the elephant. I think the same thing occurs in the human teeth. The difficulty is in our mode of life. But I have no doubt that in vigorous, robust constitutions, there is recuperative power in the pulps of the teeth to effect such protection.

[To be continued.]

